# Influence of Interleaved Films on the Mechanical Properties of Carbon Fiber Fabric/Polypropylene Thermoplastic Composites

**DOI:** 10.3390/ma9050344

**Published:** 2016-05-06

**Authors:** Jong Won Kim, Joon Seok Lee

**Affiliations:** 1 Regional Research Institute for Fiber & Fashion Materials, Yeungnam University, Gyeongsan 712-749, Korea; kjwfiber@ynu.ac.kr; 2 Department of Textile Engineering and Technology, Yeungnam University, Gyeongsan 712-749, Korea

**Keywords:** interleaved film, thermoplastic composite, polypropylene, surplus resin, compression molding

## Abstract

A laminated composite was produced using a thermoplastic prepreg by inserting an interleaved film with the same type of matrix as the prepreg during the lay-up process to improve the low interlaminar properties, which is a known weakness of laminated composites. Carbon fiber fabric (CFF) and polypropylene (PP) were used to manufacture the thermoplastic prepregs. Eight prepregs were used to produce the laminated composites. Interleaved films with different thicknesses were inserted into each prepreg. The physical properties of the composite, such as thickness, density, fiber volume fraction (*V*_f_), and void content (*V*_c_), were examined. The tensile strength, flexural strength, interlaminar shear strength (ILSS), impact property, and scanning electron microscopy (SEM) were used to characterize the mechanical properties. Compared to the composite without any inserted interleaved film, as the thickness of the inserted interleaved resin film was increased, *V*_c_ decreased by 51.45%. At the same time, however, the tensile strength decreased by 8.75%. Flexural strength increased by 3.79% and flexural modulus decreased by 15.02%. Interlaminar shear strength increased by 11.05% and impact strength increased by 15.38%. Fracture toughness of the laminated composite was improved due to insertion of interleaved film.

## 1. Introduction

Carbon fiber-reinforced polymer composites with high mechanical properties and light weight have been used widely in many engineering fields. In recent times, continuous fiber reinforced thermoplastic (CFRTP) composites have been used more widely because of their many advantages, such as fracture toughness and damage tolerance, ease of shape forming before consolidation, significantly faster manufacturing, longer shelf life of the raw material, and the ability to be reshaped and reused. These advantages are based mostly on the intrinsic properties of thermoplastic polymers [1]. According to the development of high performance polymers, such as polyetheretherketone (PEEK), polyethersulphone (PES), polyphenylene sulphide (PPS), polyethyleneterephthalate (PET), polycarbonate (PC), polyamide (PA) and polypropylene (PP), the so-called engineering plastics show significant mechanical properties [2]. In particular, a high flexural strength, high thermal stability, ease of processing, resistance to corrosion, low density, and low price has made PP one of the more promising materials as a matrix among the various thermoplastic polymers available [3,4].

Prepreg materials, in which the reinforcing fibers are pre-impregnated with resin, are commonly used to produce thermoplastic composites. The quality of the prepreg can have a significant effect on the mechanical performance of the composite. Three of the most widely used prepregging techniques are film prepregging, hot melt prepregging and solution dip prepregging [5]. In the case of film prepregging, the pressure-temperature history of the polymer dominates the impregnation quality (void content). For melting impregnation, however, this is impractical for certain polymers because of their limited tolerance to the temperatures necessary for viscosity reduction. The thermal degradation determined by a molecular weight reduction could be initiated within a few degrees of the melt temperature making the viscosity reduction difficult or impractical [6]. In addition, the use of large pressures to impregnate the polymer film into the fiber has mechanical limitations.

Considerable research has been done to discover practical methods to produce composite parts using thermoplastic prepregs. Currently, most promising processing methods include compression molding, tape winding, tape laying, braiding, and pultrusion [7]. Interlayer delamination tends to take place when two-dimensional laminated composites using film pregregs are subject to impact and bending compression [8]. Consequently, they have relatively poor through-thickness mechanical properties compared to that in the fiber direction. This is because the through-thickness mechanical properties are carried predominantly by the resin matrix. Delamination can be a most serious problem because it can reduce the mechanical properties and is difficult to detect visually [9]. Interlaminar properties of two-dimensional laminated composites can be enhanced by toughening the matrix [10], insertion of an interleaf layer [11,12], reinforcing with three-dimensional braided or woven fabrics [13], stitching of reinforcements [14], reinforcing with felts [15], and the insertion of fillers, such as carbon nanotubes, into the matrix [16,17]. In other research on the insertion of interleaved film layers, either different polymers [18,19] or polymer films, consisting of the same polymer but with different properties [20,21,22,23], have simply been inserted during the forming process. Therefore, this study examined the effects of the thickness of an interleaved film made of the same polymer film as the matrix material on the mechanical properties of laminated composites using thermoplastic prepreg.

## 2. Experimental Section

### 2.1. Materials

Woven fabric (SNC1242R, Plain, density (counts/25 mm) = 6.4, weight = 420 g/m^2^, Seanal Tech-tex Co., Gumi, Korea) using carbon fiber (Toray T-300, 12K, Tokyo, Japan) was used as the reinforcing material. For the matrix, 20 µm PP film (SBF-110, *ρ* =0.9 g/cm^3^, tensile strength = 75 MPa, Samyoung Chemical Co., Seoul, Korea) was used, and the PP film with the same thickness was also used as the interleaved film layer.

### 2.2. Preparation of the Prepreg and Laminated Composite

**Figure 1:**
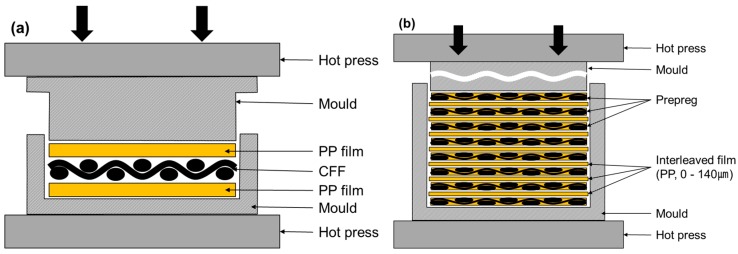
Scheme of hot press processing; (**a**) prepregs; (**b**) composites.

During the prepreg manufacturing process (Figure 1a) using carbon fiber fabric (CFF) and PP, seven PP films were laminated inside the mold (295 × 295 mm^2^) both above and below the CFF. The mold was designed to have a space between the upper and lower molds to allow surplus resin come out. After the mold was preheated inside a hot press, at a temperature of 230 °C for 10 min, the pressure was increased slowly to 28 MPa for 10 min. This is because if a high pressure is applied abruptly, the carbon fiber could be out of alignment, the resin cannot be impregnated into the fabric and the voids are difficult to squeeze out from the inter-layer. After a dwell time of 10 min, the mold was then cooled to room temperature. Subsequently, the pressure was relieved and the prepreg was then detached from the mold. The produced prepreg had a 0.45 mm thickness with a 53.81% fiber volume fraction (*V*_f_).

A similar procedure was used to prepare the laminated composite through compression molding by laminating (0°/90°) 8 prepregs, as shown in Figure 1b. The forming cycle was carried out in the same manner as prepreg production. Interleaved films were inserted between each prepreg while laminating 8 prepregs and compression molding was then carried out. The thickness of the interleaved film was varied by changing the number of interleaved films, which interleaved PP between each layer consists some of them, in the manufactured specimen. The procedure was carried out identically to the prepreg manufacturing process. As before, the pressure was increased slowly to 28 MPa to squeeze out the resin from the inter-layer.

### 2.3. Composites Characterization

The specimen thickness (mm) was averaged by measuring 5 places, including 4 edges and the center, of each 5 specimens using a thickness gauge. The actual density was determined using the Archimedes principle according to the ASTM D 792 by measuring the differences between the weight of a specimen in air and in water. The *V*_f_ of the specimen was calculated using a burn-off test according to ASTM D 2584. The specimen was inserted into a furnace in an inert environment for 10 h at 500 °C, followed by drying in a desiccator. The fiber volume fraction was then calculated based on the specimen weight ratio before and after the burn-off test. The void volume content (*V*_c_) was determined using Equation (1) below based on the ASTM D 2734 as follows:

(1) $$Vc=100-\rho_{\text{sample}}\left(\frac{{\%\text{m}}_{\text{matrix}}}{\rho_{\text{matrix}}}+\frac{{\%\text{m}}_{\text{fiber}}}{\rho_{\text{fiber}}}\right)$$

where *V*_c_ is the void volume content (%), *ρ* is the primary density and %m is the mass of each constituent.

The tensile strength, flexural strength and interlaminar shear strength (ILSS) were measured using a tensile tester (OTT-05, Oriental Co., Seoul, Korea) to determine the mechanical properties of the prepared specimens. The tensile test was performed according to the ASTM D 3039 at a crosshead speed of 2 mm/min. A three-point-bending test was carried out based on the ASTM D 790 to measure the flexural strength with a span-to-depth ratio of 16 at a crosshead speed of 1.3 mm/min. ILSS was also measured using a three-point-bending test based on the ASTM D 2344 with a span-to-depth ratio of 4 at a crosshead speed of 1 mm/min. A drop weight impact test was conducted using a drop-weight impact testing machine (Ceast 9350, Instron, Norwood, MA, USA) equipped with a 12.7 mm diameter hemispherical tip. The test was conducted with an impact velocity of 2.57 m/s^2^, 15.13 kg mass and an impact energy of 50 J. The specimen was prepared in 100 × 100 mm^2^. The cross section of prepared composite was observed by scanning electron microscopy (SEM, S-4100, Hitachi, Tokyo, Japan).

## 3. Results and Discussion

### 3.1. Thickness and Density

**Figure 2:**
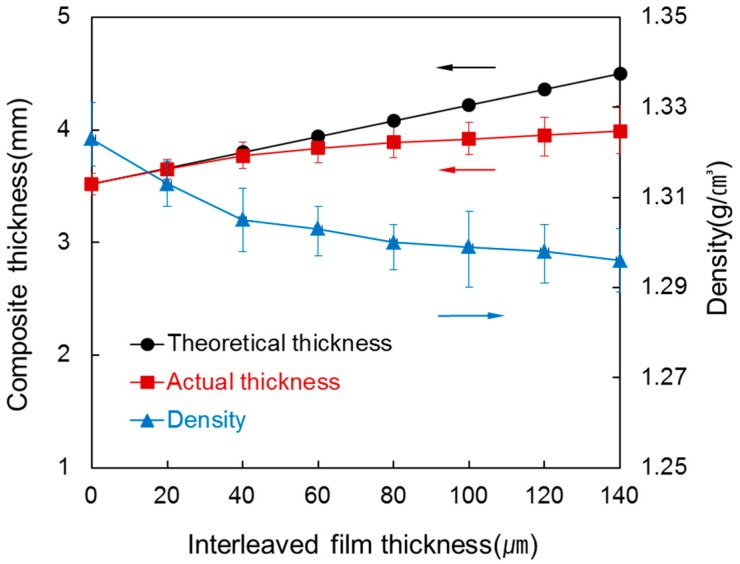
Thickness and density of the laminated composites at various interleaved film thicknesses.

Figure 2 shows the thickness and density of the prepared laminated composite determined by varying the thickness of the interleaved films between each of the 8 prepregs, ranging from 0 µm to 140 µm. The theoretical thickness is the thickness of the composite assuming that the inserted films are not squeezed out during compression molding. The actual thickness is the thickness of the composite measured after the films were squeezed out under high pressure during compression molding. Thickness of the composite without interleaved film was 3.52 (±0.11) mm. When a 20 µm interleaved film was inserted between each of the eight prepregs, the thickness of the composite was 3.65 mm which is similar to the theoretical thickness of 3.66 mm. It is because no resin is squeezed out if interleaved film is thin. However, a gap between theoretical thickness and real thickness grew as the interleaved film became thicker. It is because more surplus resin was squeezed out under high pressure. Additionally, change in the composite thickness was small over interleaved film thickness of 80 µm. Density decreased from 1.32 g/cm^3^ to 1.29 g/cm^3^ while thickness of the interleaved film increased since *V*_f_ of the composite decreased from insertion of the interleaved film. There was almost no difference in density above 80 µm because, similar to the thickness, most of the inserted interleaved films were squeezed out from the mould.

### 3.2. Fiber Volume Fraction and Void Content

**Figure 3:**
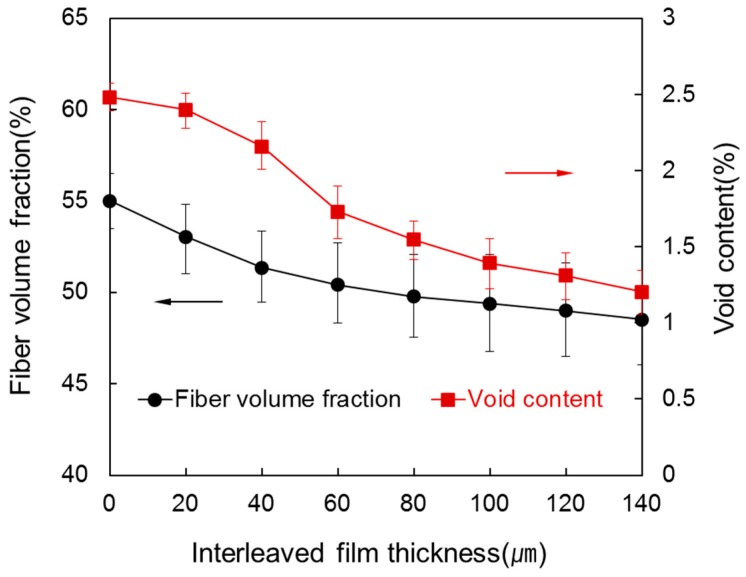
Fiber volume fraction and void content of the laminated composites at various interleaved film thicknesses.

Figure 3 shows *V*_f_ and *V*_c_ of the composite at various interleaved film thicknesses up to 140 µm between each of the eight prepregs. As thickness of interleaved film increased from 0 µm to 140 µm, *V*_f_ decreased from 55.01% to 48.52%. It is because inserted interleaved film increased the volume of composite matrix. Also, up to the film thickness of 40 µm, surplus resin remained within the composite without being squeezed out so that *V*_f_ decreased sharply. In contrast, the decrease range of V_f_ declined due to squeezed out surplus resin over 80 µm. Similar to *V*_f_, *V*_c_ decreased from 2.48% to 1.13% as thickness of the interleaved film increased. It is because, based on Equation (1), the amount of resin increased from a decrease in *V*_f_. When 20 µm interleaved film was inserted, it decreased slightly from 2.48% to 2.40%. As shown in Figure 2, it is because no surplus resin was squeezed out. However, between the film thickness of 20 µm and 80 µm, it decreased greatly from 2.40% to 1.54%. It is because surplus resin was squeezed out together with voids during the forming process under high pressure. After that, the decrease range of void was small because void content was low. Therefore, insertion of interleaved film causes squeeze out of surplus resin so that void content could decrease more or less.

### 3.3. Tensile Properties

**Figure 4:**
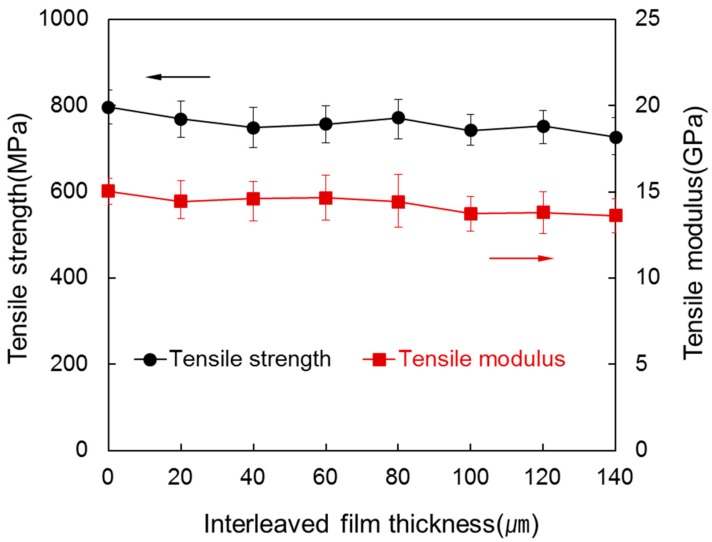
Tensile strength and tensile modulus of the laminated composites at various interleaved film thicknesses.

Figure 4 shows the tensile strength and tensile modulus of the laminated composite according to thickness of the interleaved film. The tensile strength and tensile modulus showed a tendency to decrease with increasing thickness of the interleaved film.

Tensile strength declined from 796.82 MPa to 727.04 MPa while thickness of the interleaved film increased to 140 µm. Although *V*_c_ decreases, it is mainly because of a decrease in *V*_f_. However, tensile properties were mostly lower than that of general carbon fiber composites. It seems that a small amount of air flew in during the heating process at 500 °C for 5 h under an inert environment to desize carbon fiber composite, so that the strength of the carbon fiber declined [7]. The tensile modulus showed a similar tendency to the tensile strength changing from 15.03 GPa to 13.61 GPa as the thickness of the interleaved film increased. This is because strain to break increased due to a decrease of *V*_f_.

### 3.4. Flexural Properties

**Figure 5:**
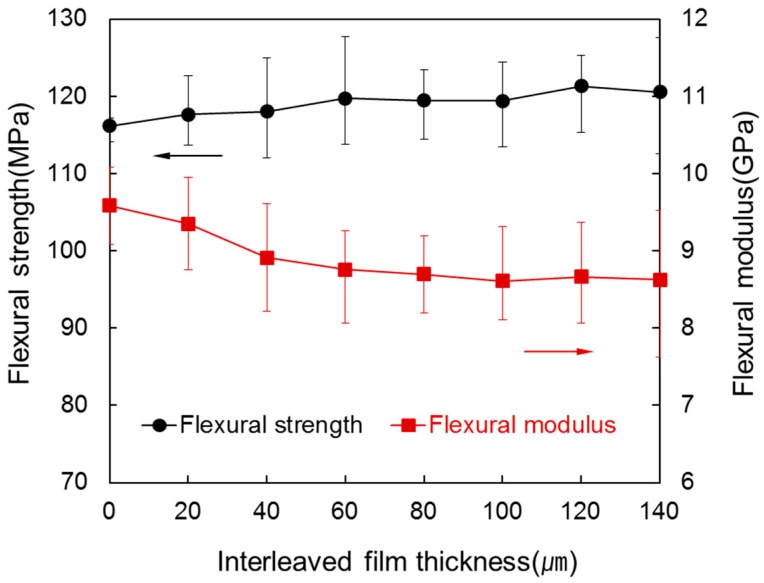
Flexural strength and flexural modulus of the laminated composites at various interleaved film thicknesses.

Figure 5 shows the flexural strength and flexural modulus of the laminated composites for various interleaved film thicknesses between each of the eight prepregs. In contrast to tensile properties, flexural strength increased slightly from 116.18 MPa to 120.58 MPa as thickness of interleaved film increased. Although *V*_f_ decreased, it is because load to break increased according to a decrease of *V*_c_ and an increase of thickness. In case of flexural modulus, it decreased greatly from 9.58 GPa to 8.14 GPa as thickness of interleaved film increased. It is because, as stiffness declined from a decrease of *V*_f_, displacement increased more than load to break in the load-displacement curve. Above an interleaved film thickness of 80 µm, the decrease range of flexural modulus was small because *V*_f_ hardly changes here. Thus, a slight increase of flexural strength and a large decrease of flexural modulus show that the fracture toughness of the composite increased from insertion of interleaved film [24,25,26].

### 3.5. Interlaminar Shear Strength

**Figure 6:**
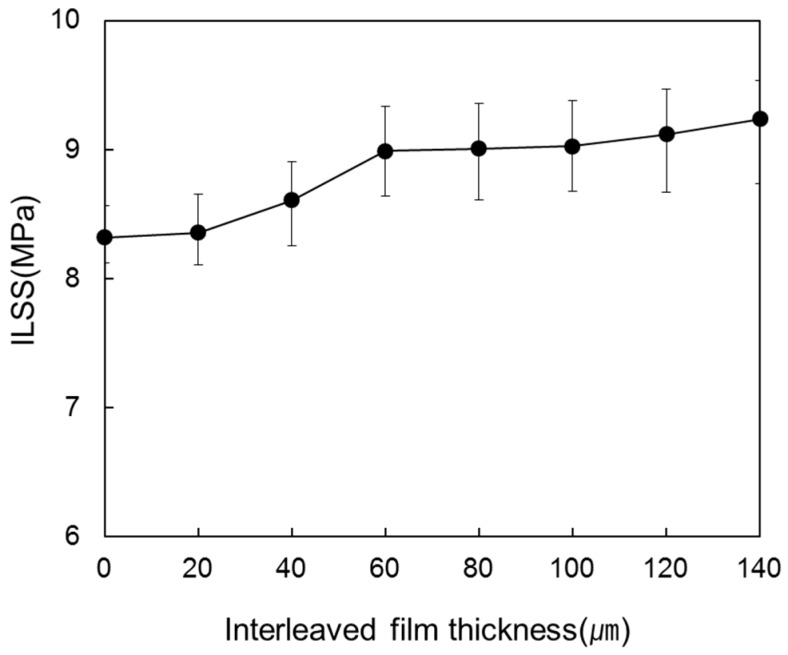
ILSS of the laminated composites at various interleaved film thicknesses.

Figure 6 shows the ILSS of the laminated composites for various interleaved film thicknesses between each of the eight prepregs. The ILSS also showed a tendency to increase as thickness of interleaved film increased. Compared to the laminated composite without an interleaved film, the ILSS increased from 8.32 GPa to 9.24 GPa when the interleaved film thickness increased to 140 µm. The reason why ILSS increased greatly seems to be that resin layer between carbon fiber layers prevented delamination.

**Figure 7:**
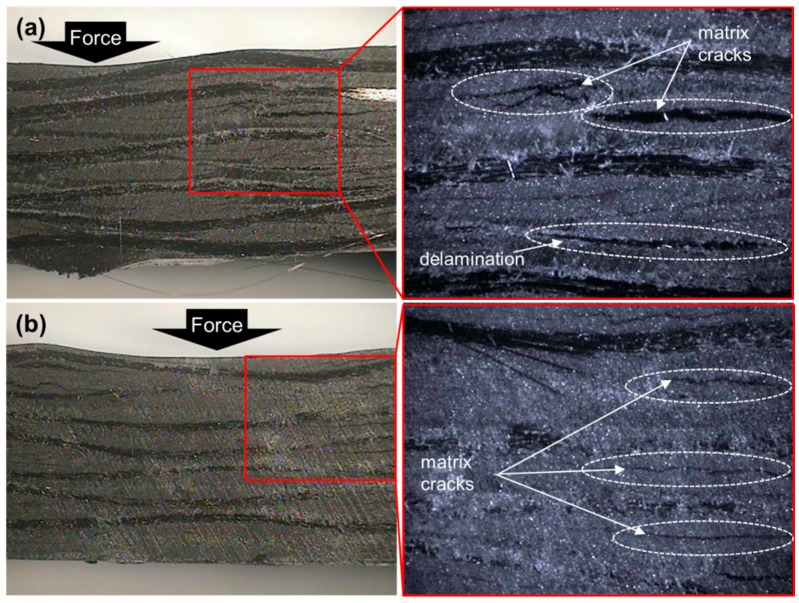
Photographs of cross sections of delamination with (**a**) no interleaved film; and (**b**) 120 µm interleaved film from ILSS test.

Figure 7 shows photographs of cross sections of the specimen after the ILSS test. As shown in Figure 7a, in case of the composite without interleaved film, matrix cracks between carbon fibers and delamination between resin layers occur. In contrast, as shown in Figure 7b, only matrix cracks between carbon fibers could be observed at interleaved film thickness of 120 µm [27].

### 3.6. Drop Weight Impact Property

**Figure 8:**
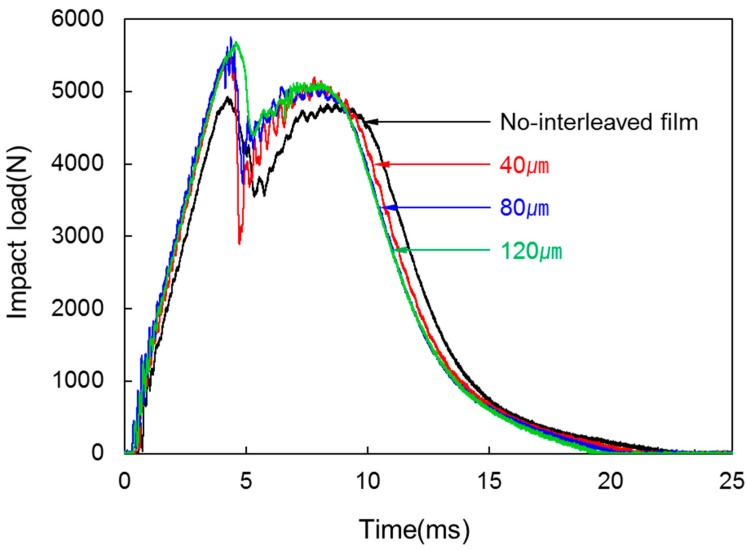
Impact load-time diagrams from the impact test of the laminated composites at various interleaved film thicknesses.

Figure 8 shows the impact load-time diagrams of the impact test of the laminated composites for various interleaved film thicknesses. The impact load tended to increase from 4932.07 N to 5690.85 N as interleaved film thickness rose to 120 µm during the forming process of laminated composites. At interleaved film thickness of 40 µm, impact load increased by 12% to 5525.14 N but there was no big difference above the thickness. Thus, there is a distinct difference in impact load between non-interleaved and interleaved. However, differences resulting from the thickness of interleaved film could not be identified. This is because, by insertion of interleaved film, existence of a resin layer improved impact resistance of the composite. Therefore, improvement in fracture toughness was shown.

**Figure 9:**
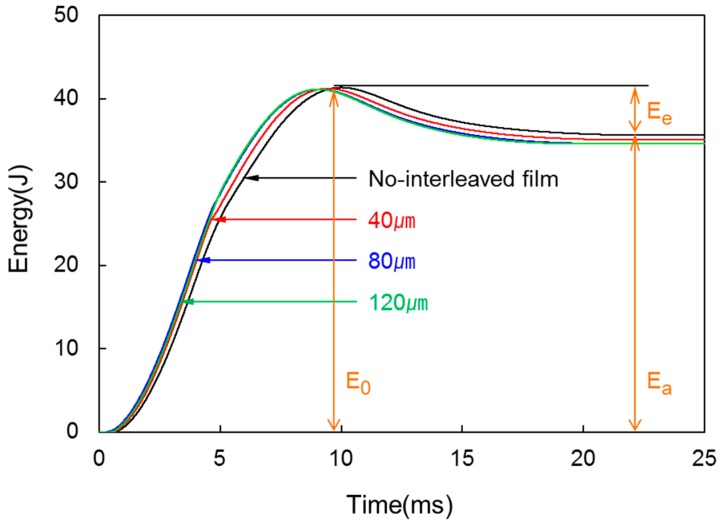
Energy-time curves of the laminated composites at various interleaved film thicknesses.

Figure 9 shows the energy-time curves from the impact test for various interleaved film thicknesses. As every curve of specimen shows, the energy begins to decrease after the maximum energy (*E*_0_). This suggests that thermoplastic composite had not been destroyed completely but absorbed the impact of low energy, and the gap between *E*_0_ and *E*_a_ is the elastic energy (*E*_e_), in which the laminated composite absorbed from deformation or vibration and was restored again to the impactor after impact. In addition, *E*_a_ decreased slightly with increasing interleaved film thickness but the decrease was not large.

**Figure 10:**
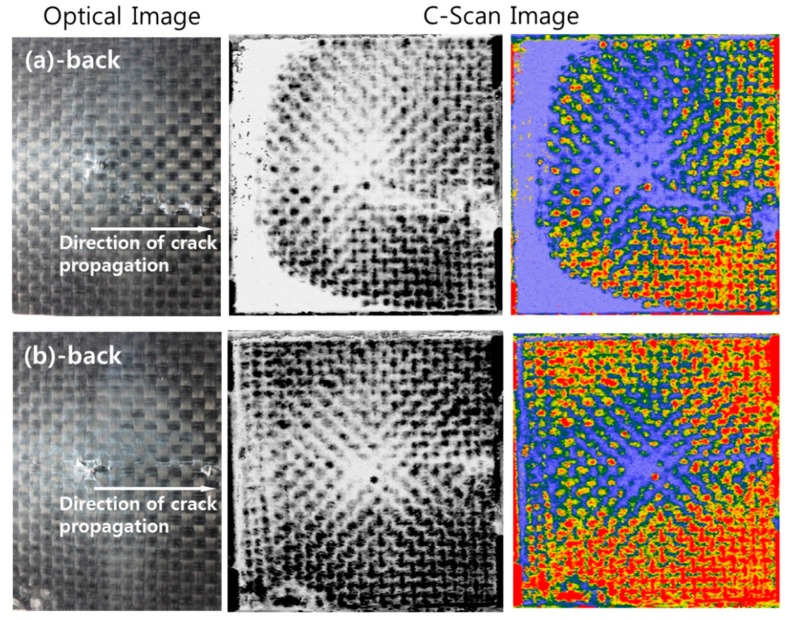
Optical images and C-Scan images of the back of the specimen after the impact test; (**a**) no interleaved film; (**b**) 60 µm interleaved film.

Figure 10 shows optical images and C-Scan images of the back of the specimen after the impact test. The laminated composites were destroyed in different ways according to whether or not there was an interleaved film layer. As shown in C-Scan images, in case of no interleaved film, damage area (blue area) of the specimen was observed in a large area and cracks that appeared from the impact were propagated to the edge. However, with interleaved film inside, damage was observed over a smaller area. It implies that interleaved film improved the impact strength of the composite [28,29,30].

**Figure 11:**
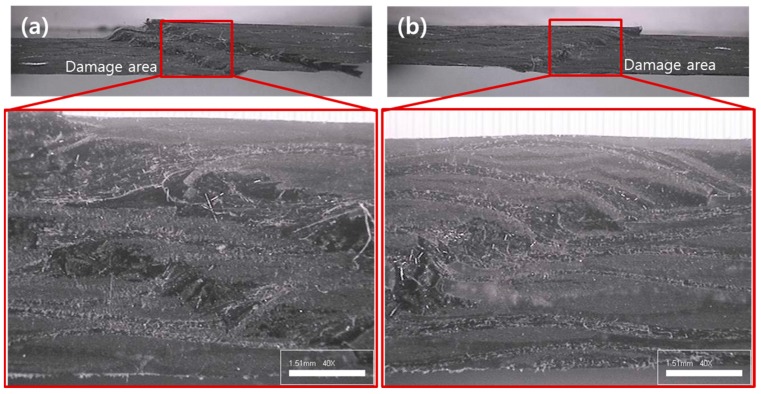
Cross section images of the specimen after the impact test; (**a**) no interleaved film; (**b**) 60 µm interleaved film.

Figure 11 shows cross section images of the specimen after the impact test. Side damage by the impact was large in case of no interleaved film (Figure 11a) but small with inserted interleaved film (Figure 11b). However, delamination could not be observed in both cases. It is because, for woven laminate, delamination initiated from the center of the impact propagated to the slope directions of warp and fill fibers [31].

### 3.7. SEM Analysis

**Figure 12:**
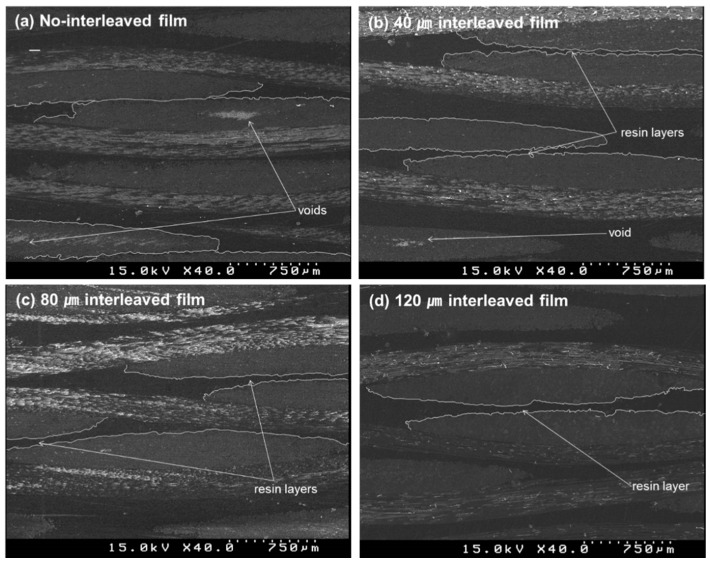
SEM images of cross sections of the laminated composites at various interleaved film thickness; (**a**) no interleaved film; (**b**) 40 µm interleaved film; (**c**) 80 µm interleaved film; (**d**) 120 µm interleaved film.

Figure 12 shows SEM images of cross sections of laminated composites for various interleaved film thicknesses between each of the eight prepregs. As shown in Figure 12a, resin layer between carbon fiber layers could not be observed when no interleaved film was inserted. If there is no resin layer, load could not be transferred from fiber to fiber so that delamination occurs due to stress concentration of resin layer. Also, voids remaining within the composite were observed since there was no squeezed out resin. As shown in Figure 12b–d, resin layers were formed between carbon fiber layers as interleaved film became thicker. These resin layers seem to prevent delamination which improved fracture toughness. Also, an increase in squeezed out resin caused a decrease in void content.

## 4. Conclusions

To manufacture laminated composite with high impregnation using a thermoplastic prepreg, PP film, which is same as the PP resin of the matrix material, was inserted as an interleaved film between each of the eight prepregs during the forming process. The mechanical properties of the laminated composite produced with different thicknesses of the interleaved film showed the following conclusions.

1. When the interleaved film thickness was increased up to 140 µm, as the amount of squeezed out surplus resin increased, the thickness of the composite specimen increased by 13.35% from 3.52 mm to 3.99 mm. The density decreased by 2.04% from 1.32 g/cm^3^ to 1.29 g/cm^3^. In addition, there was an 11.78% decrease in the fiber volume fraction from 55.01% to 48.52% and a 51.45% decrease in void content from 2.48% to 1.20%.
2. Tensile strength decreased by 8.75% from 796.82 MPa to 727.04 MPa, flexural strength slightly increased by 3.79% from 116.15 MPa to 120.58 MP and flexural modulus decreased greatly by 10.03% from 9.58 GPa to 8.62 GPa. ILSS increased by 11.06% from 8.32 MPa to 9.24 MPa. Impact load increased by 15.38% from 4932.07 N to 5690.85 N.
3. In the case of the composite with inserted interleaved film, *V*_f_ decreases and then tensile strength, tensile modulus and flexural modulus decreased so that stiffness declined. However, fracture toughness improved due to an increase in ILSS and impact properties.

## References

[1] Morgan P. (2005). Carbon Fibers and Their Composites.

[2] EL-Dessouky H.M., Lawrence C.A. (2013). Ultra-lightweight carbon fibre/thermoplastic composite material using spread tow technology. Compos. Part B.

[3] Hamada H., Fujihara K., Harada A. (2000). The influence of sizing conditions on bending properties of continuous glass fiber reinforced polypropylene composites. Compos. Part A.

[4] Han S.H., Oh H.J., Kim S.S. (2014). Evaluation of fiber surface treatment on the interfacial behavior of carbon fiber-reinforced polypropylene composites. Compos. Part B.

[5] Russo P., Acierno D., Simeoli G., Iannace S., Sorrentino L. (2013). Flexural and impact response of woven glass fiber fabric/polypropylene composites. Compos. Part B.

[6] Goodman K.E., Loos A.C. (1990). Thermoplastic prepreg manufacture. J. Thermoplast. Compos. Mater..

[7] Long A.C. (2005). Design and Manufacture of Textile Composites.

[8] Peltonen P., Lahteenkorva K., Paakkonen E.J., Jarvela P.K., Törmälä P. (1992). The influence of melt impregnation parameters on the degree of impregnation of a polypropylene/glass fiber prepreg. J. Thermoplast. Compos. Mater..

[9] Kim J., Shioya M., Kobayashi H., Kaneko J., Kido M. (2004). Mechanical properties of woven laminates and felt composites using carbon fibers. Part 1: In-plane properties. Compos. Sci. Technol..

[10] Saez S.S., Barbero E., Zaera R., Navarro C. (2005). Compression after impact of thin composite laminates. Compos. Sci. Technol..

[11] Sela N., Ishai O. (1989). Interlaminar fracture toughness and toughening of laminated composite materials: A review. Composites.

[12] Soutis C. (2005). Fibre reinforced composites in aircraft construction. Prog. Aerosp. Sci..

[13] Gao C., Yu L., Liu H., Chen L. (2012). Development of self-reinforced polymer composites. Prog. Polym. Sci..

[14] Mouritz A., Leong K., Herszberg I. (1997). A review of the effect of stitching on the in-plane mechanical properties of fiber-reinforced polymer composites. Compos. Part A.

[15] Dransfield K., Baillie C., Mai Y. (1994). Improving the delamination resistance of CFRP by stitching—A review. Compos. Sci. Technol..

[16] Karbhari V., Locurcio A. (1997). Progressive crush response of hybrid felt/fabric composite structures. J. Reinf. Plast. Comp..

[17] Wicks S., Villoria R.G., Wardle B.L. (2010). Interlaminar and intralaminar reinforcement of composite laminates with aligned carbon nanotubes. Compos. Sci. Technol..

[18] Garcia E.J., Wardle B.L., Hart A.J. (2008). Joining prepreg composite interfaces with aligned carbon nanotubes. Compos. Part A.

[19] Sohn M.S., Hu X.Z., Kim J.K., Walker L. (2000). Impact damage characterisation of carbon fibre/epoxy composites with multi-layer reinforcement. Compos. Part B.

[20] Duarte A., Herszberg I., Paton R. (1999). Impact resistance and tolerance of interleaved tape laminates. Compos. Struct..

[21] Hine P.J., Ey R.H., Ward I.M. (2008). The use of interleaved films for optimising the production and properties of hot compacted, self reinforced polymer composites. Compos. Sci. Technol..

[22] Taketa I., Ustarroz J., Gorbatikh L., Lomov S.V., Verpoest I. (2010). Interply hybrid composites with carbon fiber reinforced polypropylene and self-reinforced polypropylene. Compos. Part A.

[23] Swolfs Y., Crauwels L., Van Breda E., Gorbatikh L., Hine P., Ward I., Verpoest I. (2014). Tensile behaviour of intralayer hybrid composites of carbon fibre and self-reinforced polypropylene. Compos. Part A.

[24] Kishi H., Kuwata M., Matsuda S., Asami T., Murakami A. (2004). Damping properties of thermoplastic-elastomer interleaved carbon fiber-reinforced epoxy composites. Compos. Sci. Technol..

[25] Matsuda S., Hojo M., Ochiai S., Murakami A., Akimoto H., Ando M. (1999). Effect of ionomer thickness on mode Ι interlaminar fracture toughness for ionomer toughened CFRP. Compos. Part A.

[26] Tanimoto T. (2002). Interleaving methodology for property tailoring of CFRP laminates. Compos. Interface.

[27] Yadav S.N., Kumar V., Verma S.K. (2006). Facture toughness behavior of carbon fibre epoxy composite with Kevlar reinforced interleave. Mater. Sci. Eng. B Adv..

[28] Sarasini F., Tirillo J., Valente M., Valente T., Cioffi S., Iannace S., Sorrentino L. (2013). Effect of basalt fiber hybridization on the impact behavior under low impact velocity of glass/basalt woven fabric/epoxy resin composites. Compos. Part A.

[29] Yasaee M., Bond I.P., Trask R.S., Greenhalgh E.S. (2012). Damage control using discrete thermoplastic film inserts. Compos. Part A.

[30] Yasaee M., Killock C., Hartley J., Bond I.P. (2015). Control of compressive fatigue delamination propagation of impact damaged composites using discrete thermoplastic interleaves. Appl. Compos. Mater..

[31] Reis P.N.B., Ferreira J.A.M., Santos P., Richardson M.O.W., Santos J.B. (2012). Impact response of Kevlar composites with filled epoxy matrix. Compos. Struct..

